# WWP2 is overexpressed in human oral cancer, determining tumor size and poor prognosis in patients: downregulation of WWP2 inhibits the AKT signaling and tumor growth in mice

**DOI:** 10.18632/oncoscience.101

**Published:** 2014-11-28

**Authors:** Chonji Fukumoto, Dai Nakashima, Atsushi Kasamatsu, Motoharu Unozawa, Tomomi Shida-Sakazume, Morihiro Higo, Katsunori Ogawara, Hidetaka Yokoe, Masashi Shiiba, Hideki Tanzawa, Katsuhiro Uzawa

**Affiliations:** ^1^ Department of Oral Science, Graduate School of Medicine, Chiba University, Inohana, Chuo-ku, Chiba, Japan; ^2^ Department of Dentistry and Oral-Maxillofacial Surgery, Chiba University Hospital, Inohana, Chuo-ku, Chiba, Japan; ^3^ Department of Oral and Maxillofacial Surgery Research Institute, National Defense Medical College Hospital, Tokorozawa, Japan; ^4^ Department of Medical Oncology, Graduate School of Medicine, Chiba University, Inohana, Chuo-ku, Chiba, Japan

**Keywords:** WWP2, oral squamous carcinoma, tumoral growth, PTEN/PI3K/AKT pathway, cell cycle

## Abstract

The WW domain containing E3 ubiquitin protein ligase 2 (WWP2) encodes a member of the Nedd4 family of E3 ligases, which catalyzes the final step of the ubiquitination cascade. WWP2 is involved in tumoral growth with degradation of the tumor suppressor phosphatase and tensin homologue deleted on chromosome TEN (PTEN). However, little is known about the mechanisms and roles of WWP2 in human malignancies including oral squamous cell carcinomas (OSCCs). We found frequent WWP2 overexpression in all OSCC-derived cell lines examined that was associated with cellular growth by accelerating the cell cycle in the G1 phase via degradation of PTEN and activation of the PI3K/AKT signaling pathway. Our *in vivo* data of WWP2 silencing showed dramatic inhibition of tumoral growth with increased expression of PTEN. Our 104 primary OSCCs had significantly higher expression of WWP2 than their normal counterparts. Moreover, among the clinical variables analyzed, enhanced WWP2 expression was correlated with primary tumoral size and poor prognosis. These data suggested that WWP2 overexpression contributes to neoplastic promotion via the PTEN/PI3K/AKT pathway in OSCCs. WWP2 is likely to be a biomarker of tumoral progression and prognosis and a potential therapeutic target for development of anticancer drugs in OSCCs.

## INTRODUCTION

Considerable evidence has suggested that unexpected increases in cellular proliferation occur due to disruption of the cell-cycle control. Previous studies have reported a correlation between cell-cycle regulation and tumoral progression in human oral squamous cell carcinomas (OSCCs) [1-7]. Despite accumulating data, the precise mechanism and clinical benefit of cell-cycle regulation in OSCCs has not been determined.

The ubiquitin-proteasome pathway is a principal protein-homeostasis mechanism that controls protein quality [8-11]. A well-studied function of ubiquitination is its role in protein degradation by which polyubiquitinated proteins are recognized by proteasome and degraded rapidly. Protein ubiquitination involves a cascade of events catalyzed sequentially by ubiquitin-activating enzyme (E1), ubiquitin-conjugating enzyme, and ubiquitin ligase (E3). In addition, there is increasing evidence that aberrant expression of the enzymes involved in regulation of the ubiquitination process plays an important role in malignant transformation [12, 13].

Recently, some reports have highlighted novel functional roles for WW domain containing E3 ubiquitin protein ligase 2 (WWP2) [14-19]. WWP2 encodes a member of the Nedd4 family of E3 ligases, which catalyzes the final step of the ubiquitination cascade. Importantly, WWP2 has been proposed to be an oncoprotein in prostate cancer by targeting the phosphatase and tensin homologue deleted on chromosome TEN (PTEN) for proteasomal degradation [20-22].

PTEN is a well-defined tumor-suppressor gene with mutation or down-regulation in almost all major human cancers [23-27]. We previously also identified PTEN deregulation in OSCCs [28]. PTEN negatively regulates the PI3K/AKT signaling pathway by dephosphorylating PIP3 and thereby counteracting AKT activation [29-32]. Activated AKT controls several cellular functions such as cellular survival and death by modulating the function of numerous downstream substrates.

However, little is known about the direct mechanisms and roles between WWP2 and PTEN in human malignancies including OSCCs. We report the aberrant expression of WWP2 in OSCCs and its comprehensive analysis via the PTEN/PI3K/AKT signaling pathway. The data suggested that WWP2 functionally and clinically contributes to tumoral progression and poor prognosis in OSCCs.

## RESULTS

### Evaluation of WWP2 expression in OSCC-derived cell lines

**Figure 1:**
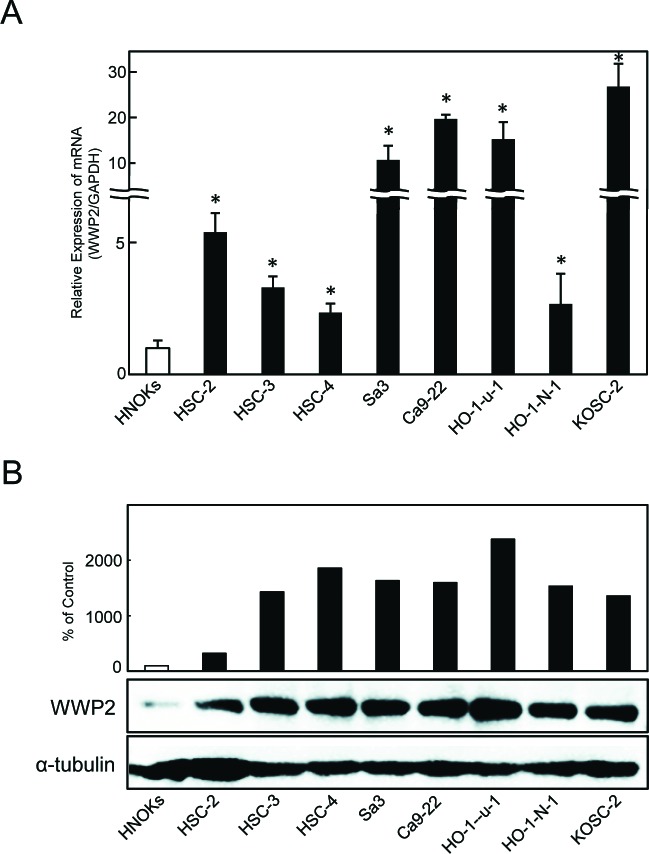
Overexpression of WWP2 in OSCC-derived cell lines (A) Quantification of WWP2 mRNA expression in OSCC-derived cell lines by qRT-PCR analysis. Eight OSCC-derived cell lines have significant (*P* < 0.05, Student's t-test) up-regulation of WWP2 mRNA compared with the HNOKs. (B) Western blotting of WWP2 protein in the OSCC-derived cell lines and HNOKs. WWP2 protein expression is up-regulated in the OSCC-derived cell lines compared with that in the HNOKs. Densitometric WWP2 protein data are normalized to α-tubulin protein levels. The values are expressed as a percentage of the HNOKs.

To investigate the mRNA expression of WWP2, we performed quantitative reverse transcriptase-polymerase chain reaction (qRT-PCR) analysis using eight OSCC-derived cell lines (HSC-2, HSC-3, HSC-4, Sa3, Ca9-22, Ho-1-u-1, Ho-1-N-1, and KOSC-2) and human normal oral keratinocytes (HNOKs). WWP2 mRNA was up-regulated in all OSCC cell lines compared with that in HNOKs (Figure 1A). The data are expressed as the mean ± standard error of the mean (SEM) (*P* < 0.05). We also performed Western blot analysis to investigate the WWP2 protein expression status in the OSCC-derived cell lines and the HNOKs (Figure 1B). A significant increase in WWP2 protein expression was seen in all OSCC-derived cell lines compared with the HNOKs. These analyses indicated that both transcription and translational products of WWP2 were highly expressed in OSCC-derived cell lines.

### Establishment of WWP2 knockdown cells

**Figure 2:**
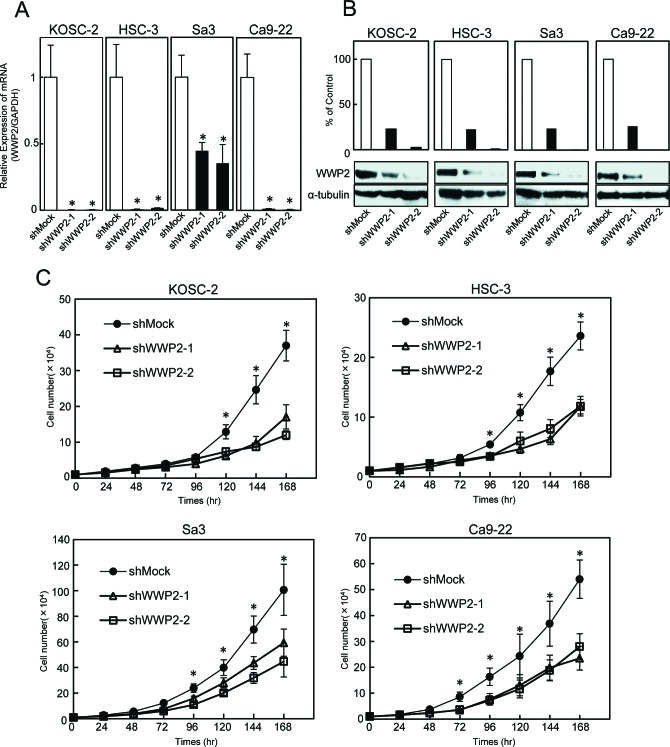
shWWP2 transfection and reduced cellular growth in shWWP2 cells (A) WWP2 mRNA levels in shWWP2-transfected cells. qRT-PCR shows that WWP2 is down-regulated significantly (*P* < 0.05, Student's t-test) in shWWP2 cells compared with shMock cells. (B) Representative Western blotting analysis and densitometric data of WWP2 protein levels in shWWP2 cells and shMock cells. WWP2 protein is decreased markedly (*P* < 0.05, Student's t-test) in shWWP2-transfected cells compared with shMock cells. Densitometric WWP2 protein data are normalized to α-tubulin protein levels. (C) Proliferation assay of the shWWP2 cells. In all cell lines, the cellular growth of shWWP2 cells is inhibited significantly (*P* < 0.05, Student's t-test) compared with shMock cells after 7 days. The results are expressed as the means ± SEM of values from six assays.

To study the possible function of WWP2 in OSCCs, the OSCC-derived cell lines, HSC-3, Sa3, Ca9-22 and KOSC-2, were transfected with WWP2 shRNA and shMock as controls. To confirm that shWWP2 transfection works and WWP2 mRNA and protein decrease, we performed qRT-PCR and Western blotting (Figure 2A and B, respectively). The expression levels of WWP2 mRNA and protein in shWWP2-transfected cells decreased significantly (*P* < 0.05) compared with the shMock-transfected cells.

### Functional analyses of WWP2 knockdown cells

We also performed cellular proliferation and migration assays to evaluate the biologic effects of shWWP2 cells. The migration assay (Supplementary Figure 1) showed that the wounds in three shWWP2-transfected cell lines (KOSC-2, HSC-3, and Ca9-22) closed at the same speed as that in the shMock cells. In Sa3, only the shWWP2-1 cells closed significantly (*P* < 0.05) later than in the mock cells. When we performed a cellular proliferation assay (Figure 2C) to evaluate the effect of WWP2 knockdown on cellular growth, we found a significant (*P* < 0.05) decrease in cellular growth in all shWWP2 cells compared with shMock cells. These assays showed that WWP2 knockdown decreased cellular growth not migration capability.

### Knockdown of WWP2 induces down-expression of PTEN and regulation of AKT-PI3K pathway

**Figure 3:**
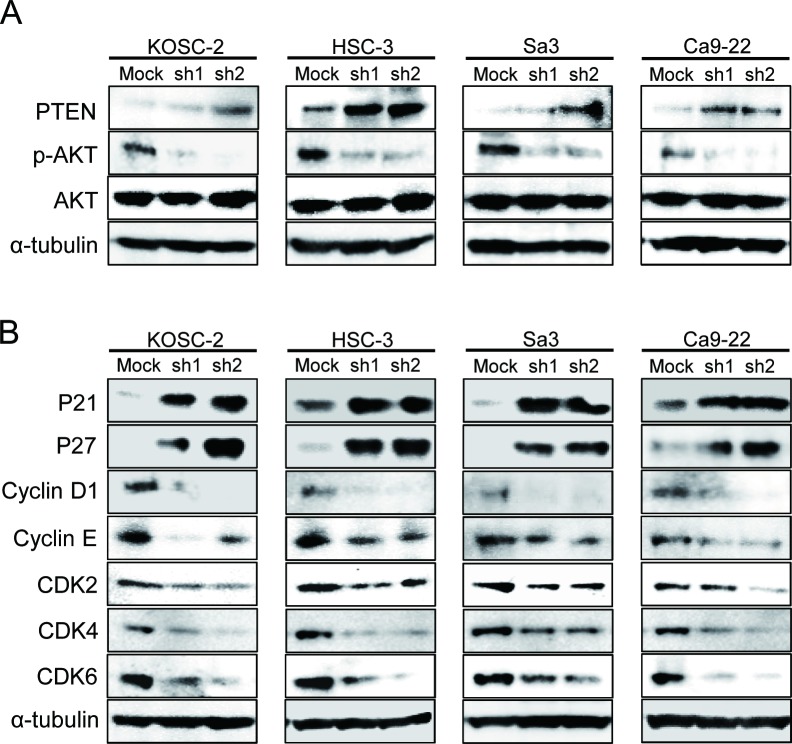
shWWP2 increases PTEN and promotes G2/M arrest via the PI3K-AKT pathway (A) Western blot analyses relevant to the PTEN/PI3K/AKT pathway in shWWP2 and shMock cells. shWWP2 cells have overexpression of PTEN and down-expression of p-AKT. Densitometric PTEN, AKT, and p-AKT protein data are normalized to α-tubulin protein levels. (B) Western blot analyses of cell-cycle-related genes in shWWP2 and shMock cells. shWWP2 cells indicate overexpression of p21^Cip1^ and p27^Kip1^ as CDK inhibitors and down-regulation of cyclin D1, cyclin E, CDK2, CDK4, and CDK6.

As a result of reducing cellular growth of the shWWP2 transfected cells, we investigated PTEN expression, reported to be associated with WWP2, following the PI3K-AKT pathway, which is activated frequently in many cancers (Figure 3A). PTEN protein increased and phosphorylated-AKT (p-AKT) protein decreased in shWWP2-transfected cells compared with shMock cells. These results suggested that the AKT signaling pathway is suppressed frequently in shWWP2-transfected cells.

### Knockdown of WWP2 induces G1 phase accumulation

**Figure 4:**
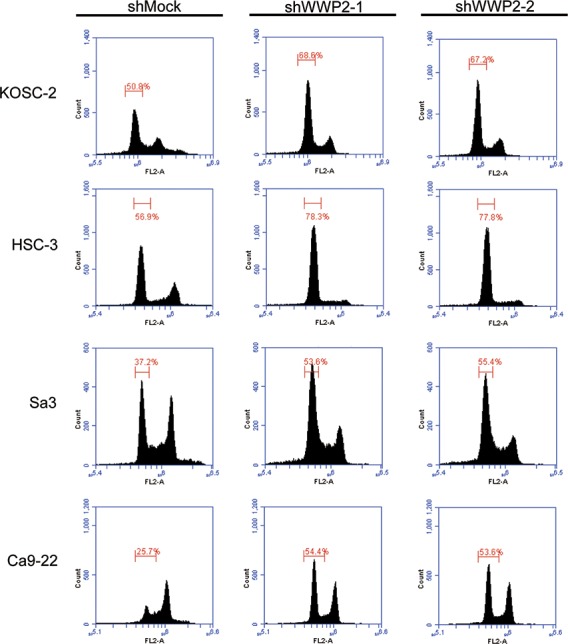
shWWP2 increases PTEN and promotes G2/M arrest via the PI3K-AKT pathway Cell-cycle analysis by flow cytometry. The percentage of the G1/S phase in shKIF4A cells is significantly (*P* < 0.05, Student's t-test) higher than in shMock cells. The data are expressed as the means ± SEM of values from three assays.

Following the activity of the PTEN and PI3K-AKT pathways, we assessed the protein expression level of cell-cycle-related genes in shWWP2-transfected cells; p21^Cip1^, p27^Kip1^, cyclin D1, cyclin E, CDK2, CDK4, and CDK6 (Figure 3B). The protein expressions of p21^Cip1^ and p27^Kip1^ were up-regulated, while cyclin D1, cyclin E, CDK2, CDK4, and CDK6 were down-regulated in all shWWP2 cells. We also investigated the cell-cycle distributions of the shWWP2-transfected cells using fluorescence-activated cell sorting (Figure 4). The arrest of cells in the G1 phase in the shWWP2-transfected cells was significantly (*P* < 0.05) higher than that in shMock cells. These data supported the previous result of cell-cycle-related gene expression and suggested that down-regulation of WWP2 inhibited cellular proliferation, which induced G1 arrest.

### Knockdown of WWP2 inhibited tumoral growth *in vivo*

**Figure 5:**
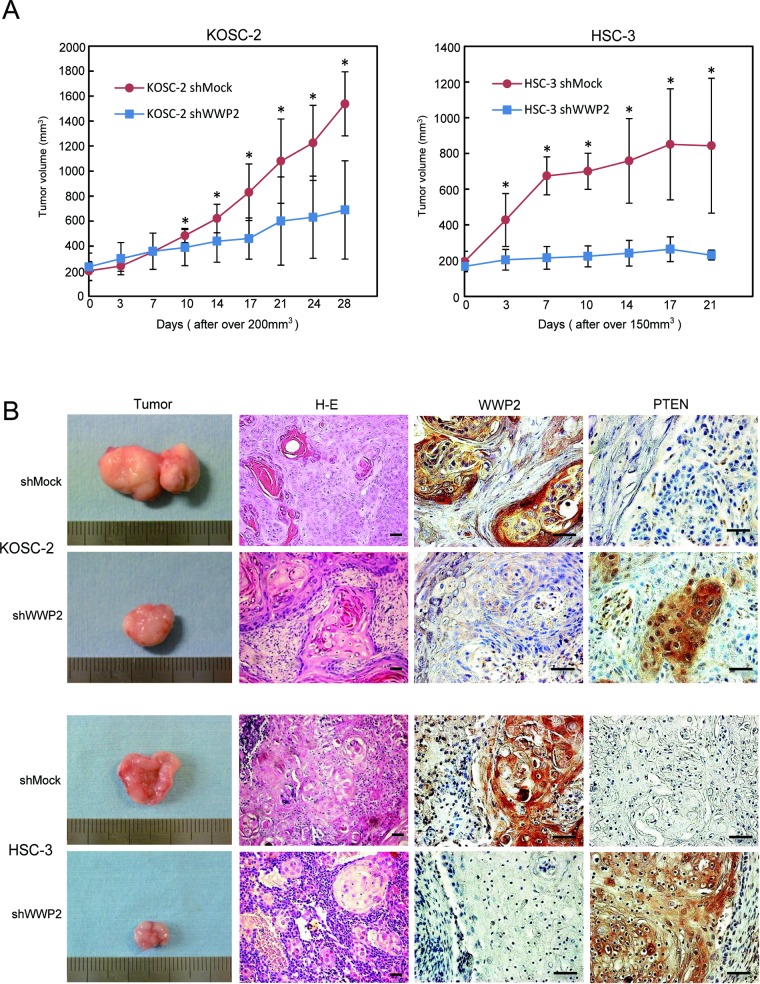
shWWP2 inhibits tumoral growth *in vivo* (A) To define the effect of WWP2 on tumoral growth *in vivo*, shWWP2 and shMock-transfected cells of two cell lines, KOSC-2 and HSC-3, were injected subcutaneously into the backs of female nude mice, respectively (3or 4 mice in each group). The *in vitro* findings showed that the mean tumoral volume of the shWWP2-transfected cells is significantly (*P* < 0.05, Student's t-test) smaller compared with the shMock-transfected. (B) Original magnification, ×200 (hematoxylin and eosin) and ×400 (WWP2 and PTEN). Scale bars, 50 μm. IHC clearly shows decreased immunostaining for WWP2 and increased immunostaining for PTEN in the shWWP2-derived tumors than in the shMock-injected mice. Hematoxylin and eosin staining confirms the presence of tumoral cells. H&E, hematoxylin and eosin.

To define the effect of WWP2 on tumoral growth *in vivo*, shWWP2 and shMock-transfected cells of two cell lines, KOSC-2 and HSC-3, were injected subcutaneously into the backs of female nude mice, respectively (3 or 4 mice in each group). According to our *in vitro* findings, the mean tumoral volume of the shWWP2-transfected cells was significantly (*P* < 0.05) smaller compared to the shMock-transfected cells (Figure 5A). Immunohistochemistry staining (IHC) of WWP2 and PTEN with tumoral tissues *in vivo* clearly showed decreased immunostaining for WWP2 and increased immunostaining for PTEN in the shWWP2-derived tumors compared with shMock injected mice (Figure 5B). These data showed that WWP2 knockdown decreased tumoral growth via increased PTEN *in vivo*.

### Evaluation of WWP2 expression in primary OSCCs and correlation with poor prognosis

**Figure 6:**
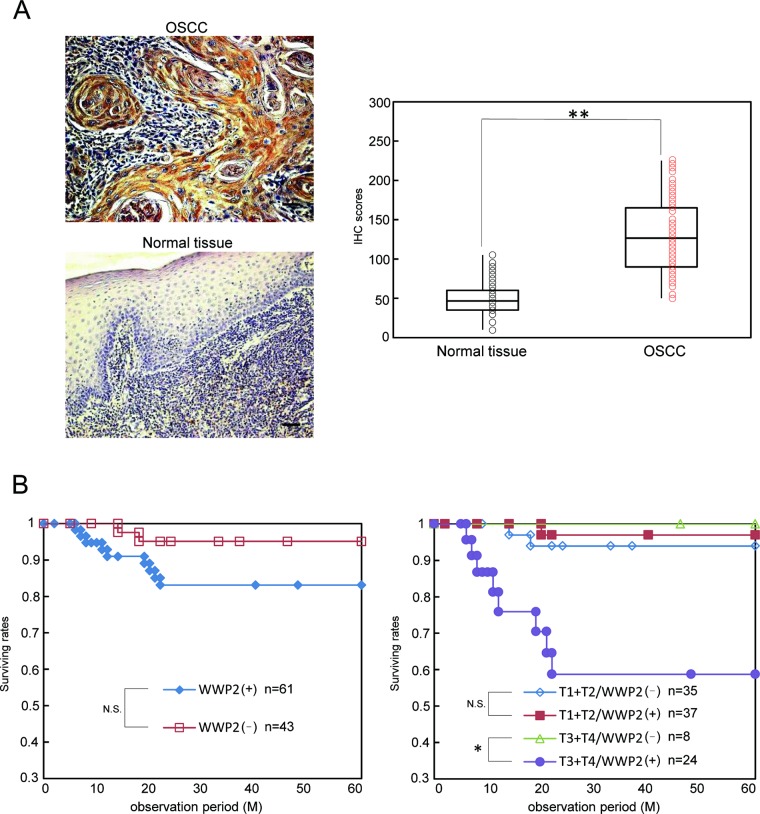
Evaluation of WWP2 protein expression in primary OSCCs and correlation with poor prognosis (A) Representative IHC results for WWP2 protein in normal oral tissue and primary OSCC. Original magnification, ×200. Scale bars, 50 μm. WWP2 is highly overexpressed in OSCCs compared to normal oral tissues. The state of WWP2 protein expression in primary OSCCs (n=104) and the normal counterparts. The WWP2 IHC scores of normal oral tissues range from 10 to 105 (median, 45.0) and OSCCs range from 50 to 225 (median, 122.5). WWP2 protein expression levels in OSCCs are significantly (*P*< 0.01, Student's t-test) higher than in normal oral tissues. (B) Five-year survival rate. The left graph shows survival rates with WWP2 expression. The survival rates in the WWP2-positive group are lower than in WWP2-negative group, but the difference does not reach significance. The right graph shows the survival rates based on the degree of primary tumoral progression, T1+T2 or T3 +T4, with the WWP2 expression. In the T1+T2 group. there is no significant difference in the survival rates between the WWP2-positive and -negative groups. In the T3+T4 group, the survival rates in the WWP2-positive group are significantly (*P* < 0.05, log-rank test) lower than in the WWP2-negative group.

To determine the expression status of WWP2 in primary OSCCs and the relation to the clinicopathological characteristics, we performed IHC of the WWP2 protein in primary OSCCs and paired normal oral tissues from 104 patients and statistical analysis using the IHC scoring system[33-38]. The WWP2 protein expression of primary OSCCs was significantly (*P* < 0.01) higher than in normal tissues (Figure 6A). Moreover the WWP2 IHC scores of the normal oral tissues ranged from 27.5 to 132.4 (median, 93.5) and those of the OSCCs ranged from 60.5 to 230.4 (median, 150.6). Table 1 shows the correlations between the clinicopathological characteristics of the patients with OSCC and the status of WWP2 protein expression. Among the clinical parameters, WWP2 expression was correlated significantly (*P* < 0.05) with the primary size of the OSCC tumors. These results suggested that WWP2 may be related closely to tumoral progression.

**Table 1:** Evaluation of WWP2 protein expression in primary OSCCs and correlation with poor prognosis

Clinical classification	Results of immunostaining No. patients (%)					
	Total	WWP2(+)		WWP2(−)		*P* value
**Age at surgery (years)**						
＜60	28	16	(57)	12	(43)	0.621
60～70	23	16	(70)	7	(30)	
70＜	53	29	(55)	24	(45)	
**Gender**						
Male	66	39	(59)	27	(41)	0.906
Female	38	22	(58)	16	(42)	
**T-primary tumor**						
1	8	4	(50)	4	(50)	0.046*
2	64	33	(52)	31	(48)	
3	15	12	(80)	3	(20)	
4	17	12	(71)	5	(29)	
1+2	72	37	(51)	35	(49)	0.025*
3+4	32	24	(75)	8	(25)	
**N-regional lymph node**						
(−)	61	39	(64)	22	(36)	0.195
(+)	43	22	(51)	21	(49)	
**Stage**						
Ⅰ	7	4	(57)	3	(43)	0.749
Ⅱ	41	24	(59)	17	(41)	
Ⅲ	17	12	(71)	5	(29)	
Ⅳ	39	21	(54)	18	(46)	
Ⅰ+Ⅱ	48	28	(58)	20	(42)	0.951
Ⅲ+Ⅳ	56	33	(59)	23	(41)	
**Histopathologic type**						
Well	62	40	(65)	22	(35)	0.109
Moderate	36	19	(53)	17	(47)	
Poor	6	2	(33)	4	(67)	
**Tumor site**						
Tongue	30	18	(60)	12	(40)	0.420
Gingiva	55	34	(62)	21	(38)	
Buccal mucosa	9	6	(67)	3	(33)	
soft palate	8	2	(25)	6	(75)	
Oral floor	2	1	(50)	1	(50)	
**Vascular invasion**						
(−)	77	45	(58)	32	(41)	0.941
(+)	27	16	(59)	11	(41)	

* *P* <0.05

The correlations between the clinicopathological characteristics of the OSCC patients and the status of the WWP2 protein expression. Among the clinical parameters, WWP2 expression was related significantly (*P* < 0.05) to the primary size of the OSCC tumors.

We graphed the 5-year survival rates to investigate whether WWP2 expression was correlated with poor prognosis. First, as a result of analyzing in all 104 patients, the survival rates in the WWP2-positive group were lower than in the WWP2-negative group, but the difference did not reach significance (Figure 4B, left graph). We then conducted an analysis based on the degree of primary tumoral progression, T1+T2 or T3 +T4, with WWP2 expression (Figure 4B, right graph). There was no significant difference in the survival rates between the WWP2-positive and -negative groups among the T1+T2 tumors, but the survival rate in the WWP2-positive group was significantly (*P* < 0.05, log-rank test) lower than in the WWP2-negative group among the T3+T4 tumors, indicating that WWP2 expression was correlated with poor prognosis.

## DISCUSSION

WWP2 is a member of the Nedd4 family of E3 ligases, which catalyzes the final step of the ubiquitination cascade[14-19]. The ubiquitin-proteasome pathway is one of the principal protein-homeostasis mechanisms that controls protein quality[8-13]. A well-studied function of ubiquitination is its role in protein degradation by which polyubiquitinated proteins are recognized by proteasomes and degraded rapidly. It has been reported that WWP2 is involved in tumoral growth with degradation of the tumoral suppressor PTEN[20-22, 39]. However, little is known about its direct mechanisms and roles in human malignancies, including OSCCs. We showed that WWP2 was overexpressed frequently in all OSCC-derived cell lines examined (Figure 1). The knockdown of WWP2 in OSCC-derived cell lines resulted in a dramatic effect on growth inhibition *in vitro* and *in vivo* via arrest of the G1/S phase (Figures 2, 3, and 4). Moreover, the expression levels of WWP2 in primary OSCCs were correlated significantly with tumoral size and poor prognosis (Figures 5, 6, and Table 1). The current data provided a novel insight into the role of aberrant WWP2 function as an oncogenic process in this disease.

The expression and function of WWP2 in OSCCs has not been investigated previously. In the current study, we found that WWP2 was up-regulated at the pretranscription and protein levels in all OSCC-derived cell lines examined (Figure 1). To investigate the possible function of WWP2, we created stable WWP2 knockdown transfectants in four OSCC-derived cell lines (Figure 2A and B). Interestingly, suppression of WWP2 significantly decreased cellular proliferation (Figure 2C), which suggested the hypothesis that WWP2 expression is necessary for oral carcinogenesis and neoplastic progression.

We then investigated if reduced cellular growth after WWP2 knockdown may be due to up-regulation of PTEN and hence decreased PI3K/AKT signaling. Several reports have suggested that WWP2 physically interacts with the tumor suppressor PTEN and mediated its degradation through an ubiquitination-dependent pathway[20, 21, 39]. Furthermore, it was recently reported that WWP2-mediated depletion of PTEN, which is a negative regulator of the PI3K/AKT signaling pathway, consequently elevated AKT signaling activity, and rendered prostate cancer cell lines resistant to stress-inducible cell mortality[21]. Based on this evidence and the current findings, we speculated that WWP2 expression is relevant to phosphorylation of AKT and activation of the PI3K/AKT signaling pathway. Indeed, the current study showed that WWP2 silencing resulted in increased PTEN protein levels and simultaneously decreased AKT phosphorylation with no significant effect on the total AKT levels (Figure 3A). The PTEN/PI3K/AKT signaling pathway is related to many cellular processes, such as proliferation and cellular survival[29, 31, 32]. Blocking AKT activation inhibits proliferation and induces apoptosis of cells of many cancer types including oral cancer[40-43]. The current findings indicated that WWP2 expression is relevant to phosphorylation of AKT and activation of the PI3K/AKT signaling pathway. In addition to decreased phosphorylation of AKT, OSCC cells with WWP2 silencing revealed cell-cycle arrest at the G1 phase with up-regulation of p21^Cip1^ and p27^Kip1^ and down-regulation of CDK complexes, such as CDK2, CDK4, CDK6, cyclin D1, and cyclin E (Figures 3B and 4). AKT is the central mediator of the AKT pathway with numerous downstream molecules that regulate cellular proliferation[41, 43]. Members of the Cip/Kip family, p21^Cip1^ and p27^Kip1^, are implicated in the negative regulation of cell-cycle progression from the G1 to S phase by binding to and modulating CDK activity[44, 45]. In contrast, CDK complexes promote progression from the G1 to S phase by triggering DNA replication and regulating genes[45-48]. AKT regulates p21^Cip1^ and p27^Kip1^ negatively and CDK complexes positively[49, 50]. These data suggested that WWP2 regulates the PTEN/PI3K/AKT signaling pathway by depleting PTEN and phosphorylating AKT and subsequently promotes the G1 cell cycle in OSCC progression.

To investigate the oncogenic potential of WWP2 *in vivo*, we tested a xenograft assay. Our *in vivo* data showed significant inhibition of xenografted tumoral growth and transformation, and overexpression of PTEN in tumoral tissues by WWP2 silencing. In this context, Maddika *et al* [21]. also reported that stable expression of WWP2 enhanced transformation of prostate cancer cells based on soft-agar colony formation assays, and enhanced tumorigenicity was observed using *in vivo* xenograft experiments, suggesting that WWP2 oncogenic potential may contribute to PTEN in human cancers including OSCCs.

In conclusion, our data supported a potential mechanism by which aberrant expression of WWP2 in OSCCs contributes to neoplastic promotion via the PTEN/PI3K/AKT pathway. This discovery may provide a novel strategy that WWP2 may be a useful biomarker of proliferation and a possible therapeutic target for developing anti-cancer drugs for human OSCCs. It is important to design clinical studies to examine the predictive relevance of WWP2 overexpression in patients with OSCC, because cases of advanced OSCC have been divided into two groups (i.e., those with favorable and unfavorable prognosis) based on the status of WWP2 expression in primary tumors.

## MATERIAL AND METHODS

### Ethics statement

The Ethical Committee of the Graduate School of Medicine, Chiba University, approved the study protocol (approval number, 236). The study was performed in accordance with the ethical standards of the Declaration of Helsinki. All patients provided written informed consent to participation in this research.

All experimental animals were treated in accordance with the guidelines of Chiba University. Experimental animals were sacrificed by cervical dislocation. We exerted the utmost effort to relieve pain associated with the study procedures. The Committee on the Ethics of Animal Experiments of Chiba University (approval number, 25–221) approved the study protocol.

### OSCC-derived cell lines and tissue samples

Immortalized human OSCC-derived cell lines (HSC-2, HSC-3, HSC-4, Sa3, Ca9-22, HO-1-u-1, HO-1-N-1, and KOSC-2) were obtained from the Human Science Research Resources Bank (Osaka, Japan) or the RIKEN BioResource Center (Ibaraki, Japan) through the National Bio-Resource Project of the Ministry of Education, Culture, Sports, Science and Technology (Tokyo, Japan). Short tandem repeat profiles confirmed cellular identity. All OSCC-derived cells were grown in Dulbecco's modified Eagle medium (Sigma-Aldrich, St. Louis, MO, USA) supplemented with 10% fetal bovine serum (Sigma) and 50 units/ml penicillin and streptomycin (Sigma). Isolated and primary-cultured human HNOKs were used as a normal tissue control [51-58]. They were isolated from healthy oral mucosal epithelial specimens from young patients at Chiba University Hospital. The HNOKs were primary cultured and maintained in Keratinocyte-SFM (Invitrogen, Life Technologies Corp., Carlsbad, CA, USA) comprised of growth supplement (Invitrogen) and 50 units/ml penicillin and streptomycin (Sigma).

One-hundred and four primary OSCC samples and patient-matched normal epithelium were obtained during surgeries performed at Chiba University Hospital. The resected tissues were fixed in 20% buffered formaldehyde solution for pathologic diagnosis and IHC staining. We performed histopathological diagnosis of each OSCC sample according to the World Health Organization criteria at the Department of Pathology of Chiba University Hospital [59]. The clinicopathological stages were determined based on the TNM classification of the International Union against Cancer [60].

### cDNA preparation

Total RNA was isolated using Trizol Reagent (Invitrogen), according to the manufacturer's instructions, and the acid guanidinium thiocyanate-phenol-chloroform extraction method. cDNA was generated by total RNA from OSCC-derived cell lines using ReverTra Ace qPCR RT Master Mix (TOYOBO Life Science, Osaka, Japan) according to the manufacturer's instructions.

### Protein preparation

The cells were washed three times with cold phosphate-buffered saline (PBS) and gently centrifuged briefly. The cellular pellets were incubated at 4°C for 30 minutes in a lysis buffer (7 M urea, 2 M thiourea, 4% w/v CHAPS, and 10 mM Tris, pH 7.4) with a proteinase inhibitor cocktail (Roche Diagnostics GmbH, Mannheim, Germany). The total protein concentration was measured using a dye-binding method based on the Bradford assay with Bio-Rad Protein Assay Dye Reagent Concentrate (Bio-Rad Laboratories, Hercules, CA, USA).

### mRNA expression analysis

Real-time qRT-PCR was performed using LightCycler 480 apparatus (Roche Diagnostics GmbH) to evaluate the expression levels of WWP2 mRNA in the eight OSCC-derived cell lines (HSC-2, HSC-3, HSC-4, Sa3, Ca9-22, HO-1-u-1, HO-1-N-1, and KOSC-2). Primers and universal probes were designed using the Universal Probe Library Assay Design Center (Roche Diagnostics GmbH), which specifies the most suitable set. The primer sequences used for qRT-PCR were: WWP2, forward, 5′- CAACTCCAT TGTCTGGATCAAA -3′; reverse, 5′- TCTCCATGTCCTGGATGAAGT -3′; and universal probe #1, and the glyceraldehyde-3-phosphate dehydrogenase (GAPDH), forward, 5′-CATCTCTGCCCCCTCTGCTGA-3′; reverse, 5′-GGATGACCTTGCCCACAGCCT-3′; and universal probe #60. The transcript amount for WWP2 was estimated from the respective standard curves and normalized to the GAPDH transcript amount determined in corresponding samples.

### Western blotting analysis

Protein extracts were electrophoresed on 4-12% Bis-Tris gel and transferred to nitrocellulose membranes (Invitrogen) and blocked for 1 hour at room temperature with Blocking One (Nacalai Tesque, Inc., Kyoto, Japan). The membranes were washed and incubated with rabbit anti-WWP2 polyclonal antibody (Aviva Systems Biology, San Diego, CA, USA), mouse anti-α-tubulin polyclonal antibody (Santa Cruz Biotechnology, Inc., Dallas, TX, USA), rabbit anti-PTEN polyclonal antibody (Abcam, Cambridge, UK), rabbit anti-AKT1/2/3 polyclonal antibody (Santa Cruz Biotechnology), rabbit anti-p-AKT1/2/3 polyclonal antibody (Santa Cruz Biotechnology), rabbit anti-cyclin D1 polyclonal antibody (Santa Cruz Biotechnology), rabbit anti-cyclin E1 polyclonal antibody (Santa Cruz Biotechnology), rabbit anti-p21^Cip1^ polyclonal antibody (Santa Cruz Biotechnology), rabbit anti-p27^Kip1^ polyclonal antibody (Cell Signaling Technology, Danvers, MA, USA), rabbit anti-CDK2 monoclonal antibody (Cell Signaling Technology), rabbit anti-CDK4 monoclonal antibody (Cell Signaling Technology), and rabbit anti-CDK6 monoclonal antibody (Cell Signaling Technology) overnight at 4°C. The membranes were washed again and incubated with anti-rabbit or mouse IgG horseradish peroxidase conjugate (Promega, Madison, WI, USA). The membranes were detected using Clarity Western ECL Substrate (Bio-Rad Laboratories) and the Western blot data were visualized by exposing the membrane to a cooled CCD camera system, Light-Capture II (ATTO, Tokyo, Japan). Signal intensities were quantitated using the CS Analyzer version 3.0 software (ATTO).

### Stable transfection of WWP2 shRNA

The four OSCC-derived cell lines (HSC-3, Sa3, Ca9-22, KOSC-2) were transfected with WWP2 shRNA (shWWP2) or control shRNA (shMock) vectors (Santa Cruz Biotechnology) with Lipofectamine LTX and Plus Reagents (Invitrogen). After transfection, the stable transfectants were isolated by the culture medium containing 1.0 μg/mL puromycin (Invitrogen). Several weeks after transfection, a small colony was viable. The resistant cell colonies were picked and transferred to six-well plates and expand gradually to 10-cm dishes. To assess the efficiency of WWP2 knockdown, we performed qRT-PCR and immunoblotting.

### Cellular growth

To evaluate the effect of WWP2 knockdown on cellular proliferation, we analyzed cellular growth in shWWP2 and shMock cells. These cells were seeded in 6-cm plates at a density of 1×10^4^ viable cells. Cell were cultured for 168 hours and counted every 24 hours. We counted cells using trypsin and hemocytometer in triplicate samples.

### Migration assay

The cells were seeded in six-well plates with 10% fetal bovine serum/Dulbecco's modified Eagle medium until a confluent monolayer formed. Using a micropipette tip, one wound was created in the middle of each plate. We incubated plates at 37°C at 5% CO_2_ with free-serum medium. The results were visualized by measuring the wound area that was free of cells using the Lenaraf220b software (http://www.vector.co.jp/soft/dl/win95/art/se312811.html). The mean value was calculated from data obtained from six wells.

### Cell-cycle analysis

The transmutants were treated with 200 ng/ml nocodazole (Sigma) for 16 hours to synchronize cells at the G2/M transition [35, 61, 62]. 20 hours after treatment of nocodazole, the cells were harvested, washed with PBS, and probed with the CycleTEST Plus DNA reagent kit (Becton-Dickinson, San Jose, CA, USA) according to the manufacturer's protocol. Flow cytometric determination of the DNA content was analyzed using the BD AccuriTM C6 Flow Cytometer (Becton-Dickinson).

### Tumorigenesis and tumoral growth *in vivo*

To investigate whether WWP2 expression contributed to tumorigenesis and tumoral growth, 2×10^7^ shWWP2- and shMock-transfected two cell lines, HSC-3 and KOSC-2, were independently injected subcutaneously into the backs of female nude mice, BALB/c-nu, purchased from Oriental Yeast Co. (Tokyo, Japan). All experimental animals were treated and cared for in accordance with institutional guidelines. The tumoral sizes were measured using a digital caliper every 3 to 4 days after injection and reached a certain volume. We used the formula 4π/3× (width/2) ^2^ × (length/2) to calculate tumoral volume. The mice were sacrificed after 42 days. Tumor tissues were fixed in 10% formalin, and 4-μm paraffin sections were prepared for immunohistochemistry staining for WWP2 and PTEN.

### Immunohistochemical staining

IHC of the 4-μm sections of paraffin-embedded OSCC clinical specimens was performed. Briefly, after deparaffinization, hydration, activation of antigen, hydrogen peroxide quenching and blocking, the clinical sections were incubated with goat anti-WWP2 (AIP2) polyclonal antibody (Santa Cruz Biotechnology) at 4°C in a moist chamber overnight.

Following treatment with the first antibody, the specimens were washed in PBS and treated with rabbit polyclonal secondary antibody to goat IgG HRP (Abcam) for 2 hours at room temperature. The slides then were treated using a peroxidase substrate kit DAB (Vector Laboratories, Burlingame, CA) and lightly counterstained with hematoxylin. These were dehydrated with ethanol, cleaned with xylene, and mounted. To quantify the status of the WWP2 protein expression in clinical samples, we used the IHC scoring systems [33-38]. In summary, the mean percentages of positive tumor cells were determined in at least three random fields in each section, and the intensity of the WWP2-immunoreaction was scored as follows: 0+, none; 1+, weak; 2+, moderate; and 3+, intense. The staining intensity and the cellular numbers were multiplied to produce a WWP2 IHC score. The clinical samples with a WWP2 IHC score exceeding 105.78 (+3 standard deviation [SD] score for normal tissue) were defined as WWP2-positive. To define WWP2 positivity or negativity, we used the ±3-SD cutoff. Statistically just 0.2% of the measurement is expected to fall outside this range, because it was unlikely to be affected by random experimental error produced by sample manipulation [63]. Two independent pathologists from Chiba University Hospital, neither of whom had any knowledge of the patients' clinical status, made these judgments. To calculate the 5-year survival rate, we surveyed each patient's life and month of death.

### Statistical analysis

Basically, the statistical significance between each measurement values was evaluated using the Student's t-test. Relationships betweenWWP2 immunohistochemical staining scores and clinicopathological profiles were evaluated using the Mann-Whitney U test, and the 5-year survival rate was evaluated using the log-rank test. *P* < 0.05 was considered statistically significant. The data are expressed as the mean ± SEM.

## SUPPLEMENTARY MATERIALS FIGURES


