# Supplementary material for: WWP2 is overexpressed in human oral cancer, determining tumor size and poor prognosis in patients: downregulation of WWP2 inhibits the AKT signaling and tumor growth in mice
Source: Oncoscience. 2014 Nov 28;1(12):807–20. doi: 10.18632/oncoscience.101 (PMC4303889; doi:10.18632/oncoscience.101)
Supplement: Supplementary file 1 [file oncoscience-01-0807-s001.pdf]

WWP2 is overexpressed in human oral cancer, determining tumor size and poor prognosis in patients: downregulation of WWP2 inhibits the AKT signaling and tumor growth in mice

Supplementary Material

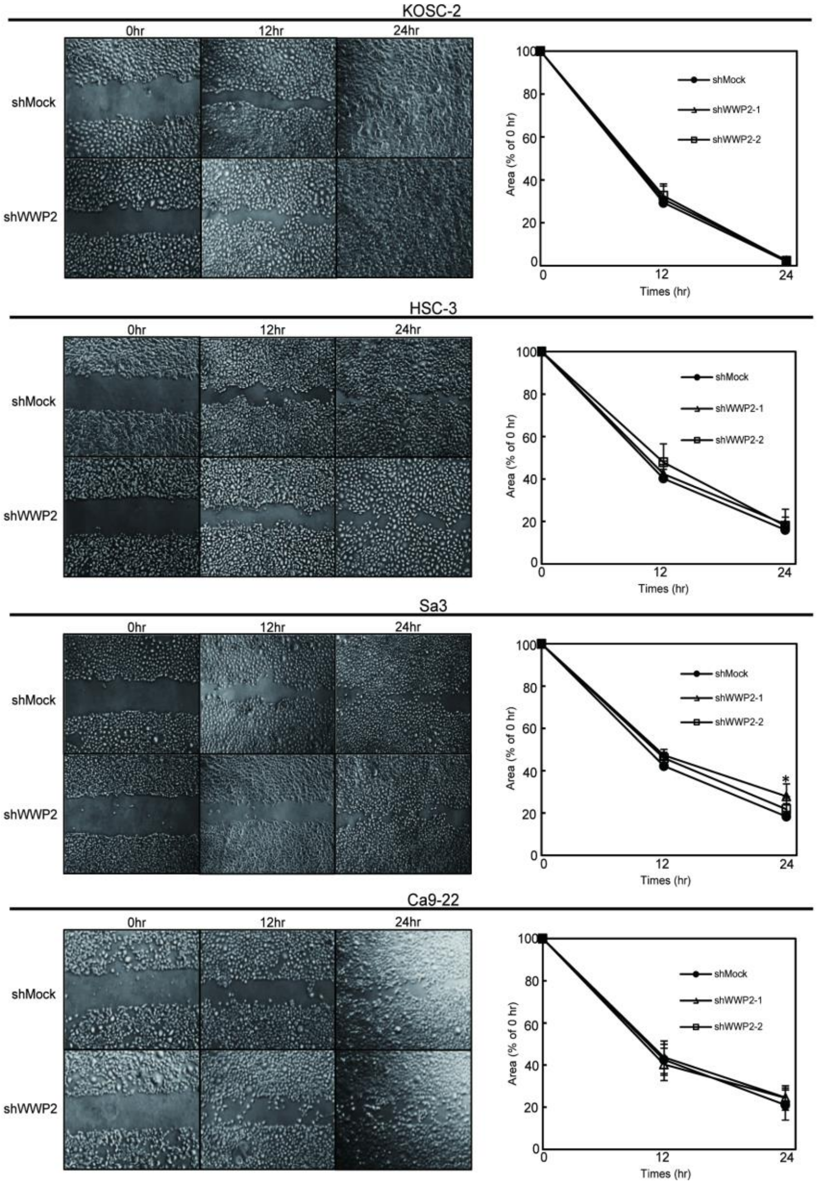

Supplementary Figure 1: Migration assay of shWWP2 cells. In three cellular lines, KOSC-2, Ca9-22, HSC-3, the wound areas have no significance between shWWP2 cells and shMock. It has

significance only in Sa3 shWWP2-1 cell on 24 hours ($p < 0.05$, Student's t-test). The results were expressed as the means ± SEM of values from six assays.

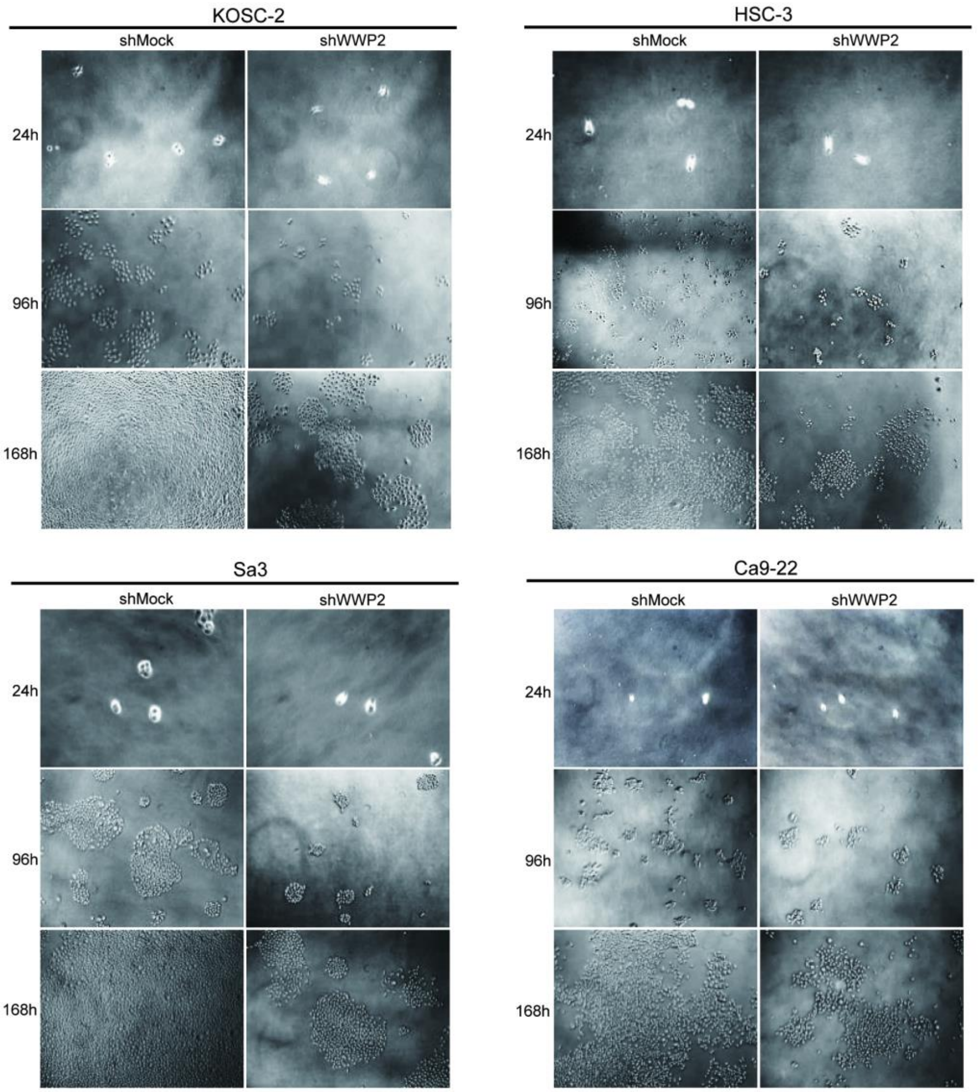

Supplementary Figure 2: Proliferation assay of the shWWP2 cells. In all cell lines, the cellular growth of shWWP2 cells is inhibited compared with shMock cells.
